# Supplementary material for: Coarse land cover datasets bias Arctic-Boreal wetland methane budgets
Source: Commun Earth Environ. 2025 Nov 14;6(1):903. doi: 10.1038/s43247-025-02963-1 (PMC12618245; doi:10.1038/s43247-025-02963-1)
Supplement: Supplementary file 2 — Supplementary information for “Coarse land cover datasets bias Arctic-Boreal wetland methane budgets” [file 43247_2025_2963_MOESM2_ESM.pdf]

Supplementary information for "Coarse land cover datasets bias Arctic-Boreal wetland methane budgets"

Josh Hashemi[1], Aleksi Räsänen[2], Tarmo Virtanen[3], Sari Juutinen[4], Guido Grosse[1,5], Mika Aurela[4], Annett Bartsch[6], Laura Chasmer[7], Scott J. Davidson[8,9], Mika Korkiakoski[4], McKenzie A. Kuhn[10], Mark J. Lara[11], Miska Luoto[12], Pekka Niittynen[13], David Olefeldt[14], Oliver Sonnentag[15], Anna-Maria Virkkala[16], Carolina Voigt[1,15,17], Claire C. Treat[1,18]

[1]Permafrost Research Section, Alfred Wegener Institute, Helmholtz Centre for Polar and Marine Research, Potsdam, Germany
[2]Geography Research Unit, University of Oulu, Oulu, Finland
[3]Ecosystems and Environment Research Programme, University of Helsinki, Helsinki, Finland
[4]Finnish Meteorological Institute, Climate System Research, Helsinki, Finland
[5]Institute of Geosciences, University of Potsdam, Potsdam, Germany
[6]b.geos GmbH, Korneuburg, Austria
[7]Department of Geography and Environment, University of Lethbridge, Canada
[8]School of Geography, Earth and Environmental Sciences, University of Plymouth, Plymouth, United Kingdom
[9]Département des sciences biologiques, Université du Québec à Montréal, Montréal, Canada
[10]Department of Geography, University of British Columbia, Vancouver, BC, Canada
[11]Department of Geography/Plant Biology, University of Illinois, Urbana, IL, United States
[12]Department of Geosciences and Geography, University of Helsinki, Helsinki, Finland
[13]Department of Environmental Sciences, University of Jyväskylä, Jyväskylä, Finland
[14]Department of Renewable Resources, University of Alberta, Edmonton, AB, Canada
[15]Département de géographie, Université de Montréal, Montréal, QC, Canada
[16]Woodwell Climate Research Centre, Falmouth, MA, United States
[17]Institute of Soil Science, Universität Hamburg, Hamburg, Germany
[18]Department of Agroecology, Aarhus University, Aarhus, Denmark

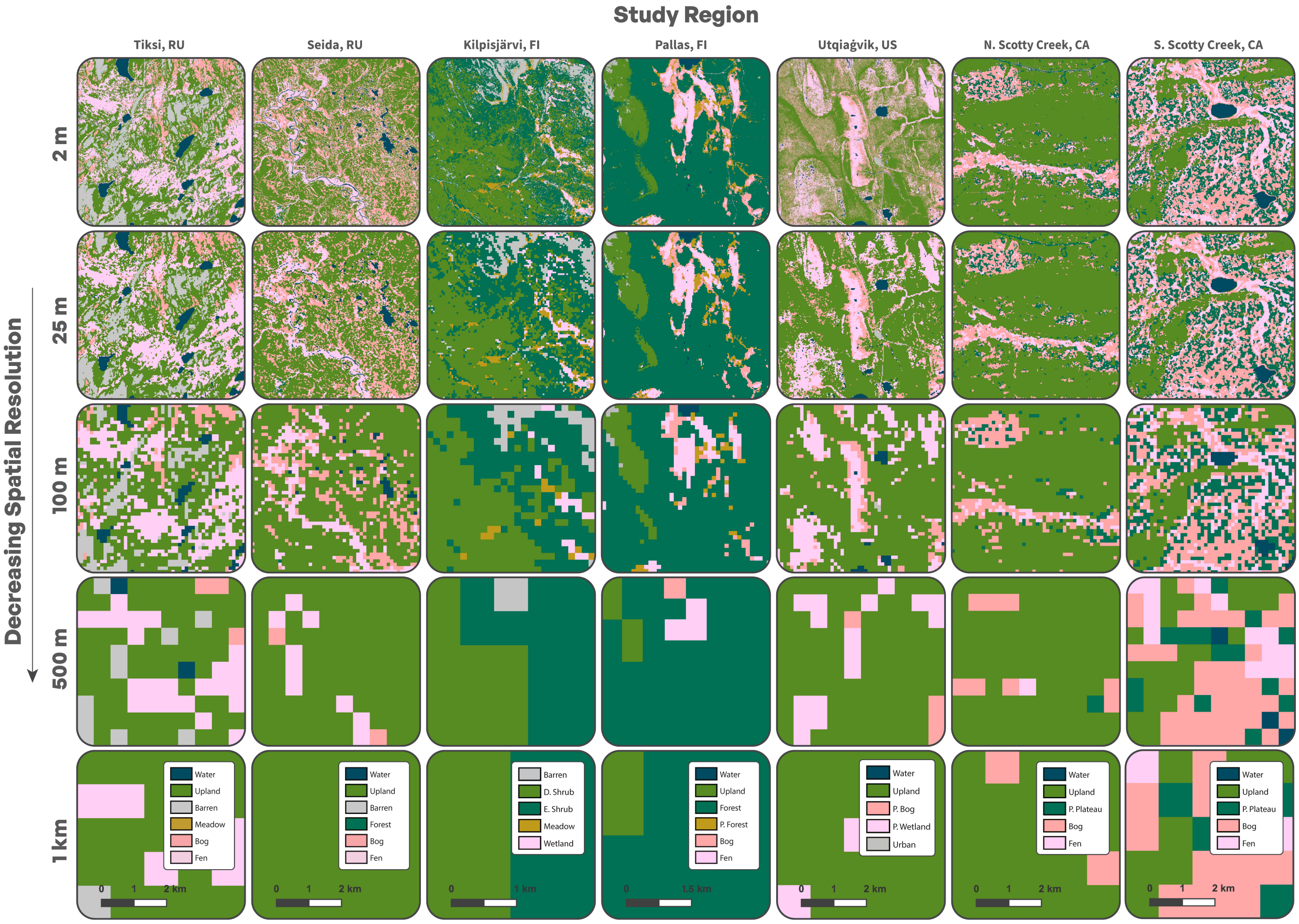

Supplementary Figure 1. Resampling from fine to coarse resolutions at each study site. Abbreviations: D. Shrub – Deciduous Shrub; E. Shrub – Evergreen Shrub; P. Bog – Permafrost Bog; P. Forest – Paludified Forest; P. Plateau – Peat Plateau; P. Wetland – Permafrost Wetland.

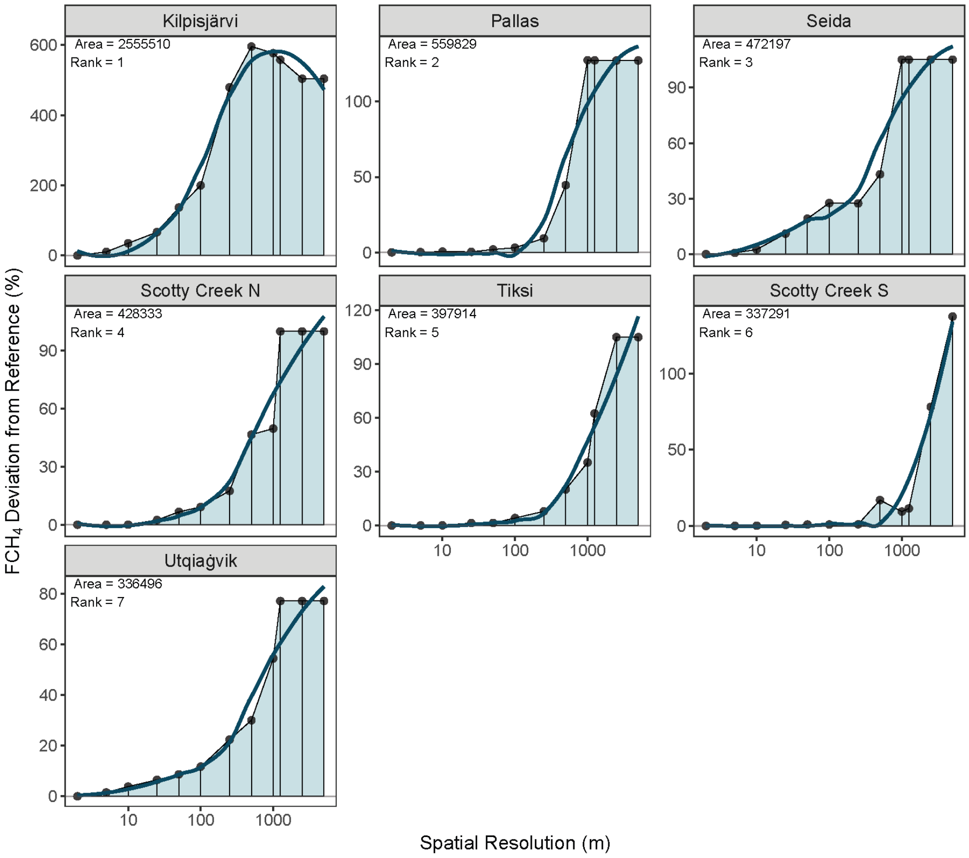

Supplementary Figure 2. LOESS-smoothed curves of FCH_4 deviation from reference across spatial resolutions for each region (n = 7). The shaded trapezoidal polygons under each curve indicate the integrated area (%· m) of percent departure across the range of resolutions, which are used to calculate resolution sensitivity scores. Spatial resolution is log-transformed on the x-axis.

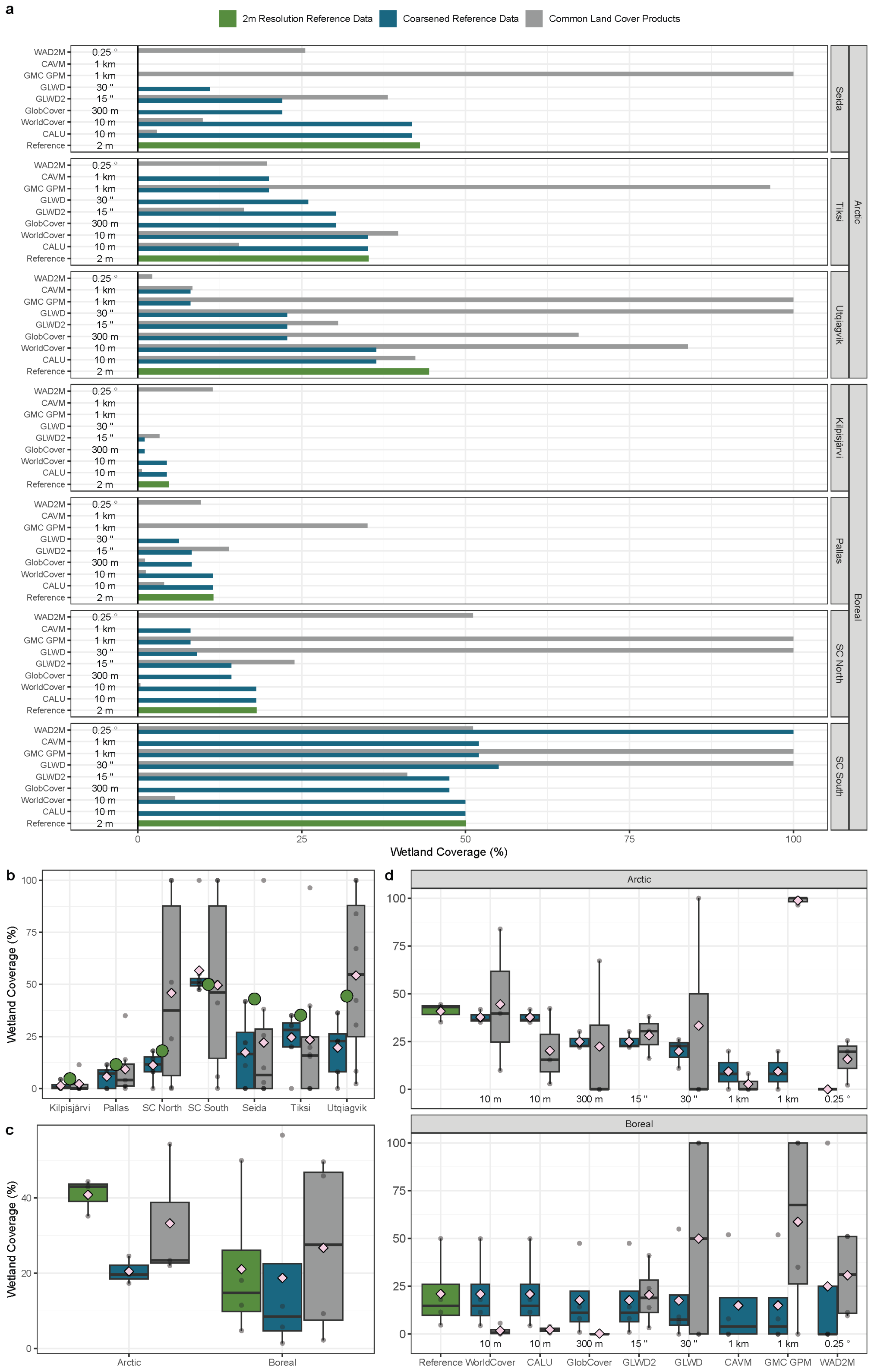

Supplementary Figure 3. Wetland coverage for each of the studied sites from commonly used land cover products (grey), from the reference maps coarsened to the closest spatial resolution step of the compared commonly used land cover product (blue), and from the reference maps at the highest spatial resolution (green) for all sites and products (a), grouped by site (b), grouped by region (c), and grouped by land cover product (d) for Arctic (upper) and boreal (lower) regions. Compared land cover products include WorldCover [1], CALU [2], GlobCover [3], GLWD2 [4], Peatmap [5], GLWD [6], CAVM (raster) [7], the Greifswald Mire Centre Global Peatland Map [8], and WAD2M [9]. Spatial resolutions are listed next to the tick label for the associated product. Pink diamonds represent means. Grey points represent underlying data. A missing bar in panel a indicates 0% wetlands for that product/spatial resolution.

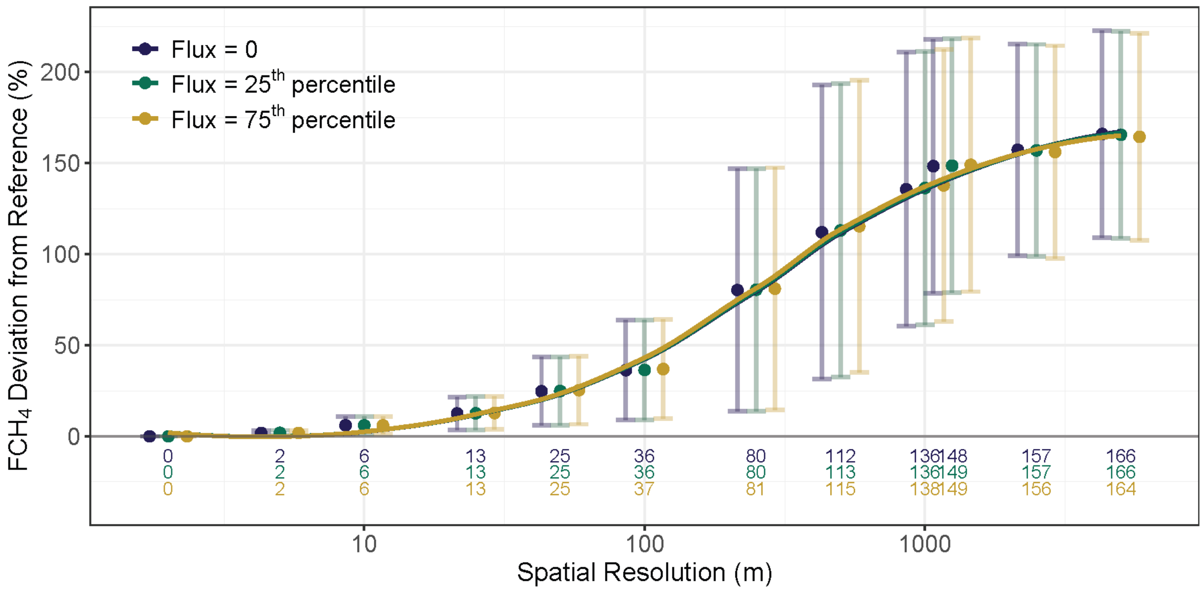

Supplementary Figure 4. Percent deviation in FCH_4 with decreasing spatial resolution with various values for inland water fluxes from the BAWLD-CH_4 database. Points and error bars represent means across sites ± standard error. Percent deviation is defined as the absolute change in FCH_4 expressed as a percentage of the FCH_4 at the nominal reference resolution. Higher values indicate larger error. Purple line (Flux=0) represents the percent deviation curve with a zero value for inland water. Blue line represents the percent deviation curve with the 25th percentile value for "medium peatland lake" as inland water flux. Yellow line represents the percent deviation curve with the 75th percentile value for "medium peatland lake" as inland water flux.

Supplementary Table 1. Information on data sources for land cover maps and methane flux measurements

Region	Land cover data source (Supp. Ref. Number)	Methane flux data source (Supp. Ref. Number)	Number of land cover classes	Number of merged land cover classes	Land cover classification data*	Resolution (m)	Dominant land cover class	Wetland coverage (%)
Utqiaġvik, USA	10,11	12	18	5	Multispectral imagery (Worldview-2)	0.5	Upland Tundra	44.4
Seida, RU	13	13	15	6	Multispectral imagery (Quickbird)	2.4	Upland Tundra	43.0
Pallas, FI	14	14	17	7	Multispectral imagery (Sentinel-2, PlanetScope), SAR, lidar	0.5	Forest	11.5
Tiksi, RU	14	15	9	6	Multispectral imagery (Quickbird, Worldview-2), Digital surface model	2	Upland Tundra	35.2
Kilpisjärvi, FI	16	16	5	5	Multispectral imagery (PlanetScope), lidar	2	Deciduous Shrub	4.7
Scotty Creek (North), CA	17	18,19	5	5	Multispectral imagery (Worldview-2), lidar	2	Upland Tundra	18.1
Scotty Creek (South), CA	17	18,19	5	5	Multispectral imagery (Worldview-2), lidar	2	Bog	50.0

*Detailed information for remote sensing datasets used to generate the reference land cover datasets can be found in the corresponding reference.

Supplementary Table 2. Fluxes and percent coverage by land cover type for each of the study regions

Region	Land cover class	Percent of landscape (%)	FCH_4 (SE) mg C m^{-2} h^{-1}
Utqiaġvik, USA	Water	2.1	1.98*
	Upland Tundra	53	0.15 (0.08)
	Barren	0.6	0.0 (0.0)
	Permafrost Bog	22.7	0.42 (0.27)
	Permafrost Wetland	21.7	1.96 (0.30)
Seida, RU	Upland Tundra	53.8	-0.05 (0.01)
	Water	2.7	0.25 (0.0)
	Forest	0.2	-2.19**
	Bog	27.8	-0.05 (0.0)
	Fen	15.2	6.94 (1.7)
	Barren	0.3	0.0 (0.0)
Pallas, FI	Upland Tundra	7.9	-0.08 (0.01)
	Water	0.7	1.98*
	Forest	74.3	-0.07 (0.0)
	Paludified Forest	4.5	0.3 (0.11)
	Bog	5.3	0.35 (0.09)
	Fen	6.2	4.33 (0.46)
	Barren	1.1	0.0 (0.0)
Tiksi, RU	Upland Tundra	44.6	-0.03 (0.01)
	Water	4.3	1.98*
	Flood Meadow	0.4	-0.01 (0.0)
	Bog	8.1	0.0 (0.0)
	Fen	27.1	2.35 (0.28)
	Barren	15.5	-0.24 (0.06)
Kilpisjärvi, FI	Barren	9.6	-0.05 (0.01)
	Deciduous Shrub	41.6	-0.1 (0.01)
	Evergreen Shrub	37.4	-0.07 (0.01)
	Meadow	6.7	-0.05 (0.01)
	Wetland	4.7	1.94 (0.66)
Scotty Creek, CA	Water	N: 0; S: 2.3	1.98*
	Upland Tundra	N: 73.6; S: 21.7	0.0 (0.0)
	Fen	N: 4.4; S: 17.9	2.5 (0.10)
	Bog	N: 13.7; S: 32.2	5.0 (1.38)
	Permafrost Plateau	N: 8.3; S: 26	0.02 (0.06)

*Values taken from BAWLD-CH_4 database [20] corresponding to median "medium-sized peatland lake". **Value taken from Olefeldt et al., 2013 [21], as reported in Treat et al., 2021 [13].

Supplementary References

1. Zanaga, D. *et al.* ESA WorldCover 10 m 2020 v100. (2021) doi:10.5281/zenodo.5571936.
2. Bartsch, A. *et al.* Circumarctic land cover diversity considering wetness gradients. *Hydrol. Earth Syst. Sci.* **28**, 2421–2481 (2024).
3. Arino, O. *et al.* Global Land Cover Map for 2009 (GlobCover 2009). PANGAEA https://doi.org/10.1594/PANGAEA.787668 (2012).
4. Lehner, B. *et al.* Mapping the world's inland surface waters: an update to the Global Lakes and Wetlands Database (GLWD v2). Preprint at https://doi.org/10.5194/essd-2024-204 (2024).
5. Xu, J., Morris, P. J., Liu, J. & Holden, J. PEATMAP: Refining estimates of global peatland distribution based on a meta-analysis. *CATENA* **160**, 134–140 (2018).
6. Lehner, B. & Döll, P. Development and validation of a global database of lakes, reservoirs and wetlands. *J. Hydrol.* **296**, 1–22 (2004).
7. Raynolds, M. K. *et al.* A raster version of the Circumpolar Arctic Vegetation Map (CAVM). *Remote Sens. Environ.* **232**, 111297 (2019).
8. Greifswald Mire Centre. Global Peatland Map 2.0. (2022).
9. Zhang, Z. *et al.* Development of the global dataset of Wetland Area and Dynamics for Methane Modeling (WAD2M). *Earth Syst. Sci. Data* **13**, 2001–2023 (2021).
10. Andresen, C., Tweedie, C. E., Villarreal, S., Cody, R. & Vargas Zesati, S. Detailed wetlands map for the Barrow Region, northern Alaska, for 2010–2013: Barrow Area Information Database (BAID). (2016).
11. Tweedie, C. E., Villarreal, S., Andresen, C. & Cody, R. Detailed wetlands map for the Barrow Region, northern Alaska, for 2010 to 2013: Barrow Area Information Database (BAID). (2016).
12. Davidson, S. J. *et al.* Vegetation Type Dominates the Spatial Variability in CH4 Emissions Across Multiple Arctic Tundra Landscapes. *Ecosystems* **19**, 1116–1132 (2016).
13. Treat, C. C. *et al.* Tundra landscape heterogeneity, not interannual variability, controls the decadal regional carbon balance in the Western Russian Arctic. *Glob. Change Biol.* **24**, 5188–5204 (2018).
14. Räsänen, A., Manninen, T., Korkiakoski, M., Lohila, A. & Virtanen, T. Predicting catchment-scale methane fluxes with multi-source remote sensing. *Landsc. Ecol.* **36**, 1177–1195 (2021).
15. Juutinen, S. *et al.* Variation in CO_2 and CH_4 fluxes among land cover types in heterogeneous Arctic tundra in northeastern Siberia. *Biogeosciences* **19**, 3151–3167 (2022).
16. Virkkala, A.-M. *et al.* High-resolution spatial patterns and drivers of terrestrial ecosystem carbon dioxide, methane, and nitrous oxide fluxes in the tundra. *Biogeosciences* **21**, 335–355 (2024).
17. Chasmer, L., Hopkinson, C., Veness, T., Quinton, W. & Baltzer, J. A decision-tree classification for low-lying complex land cover types within the zone of discontinuous permafrost. *Remote Sens. Environ.* **143**, 73–84 (2014).
18. Schulze, C. *et al.* Nitrous Oxide Fluxes in Permafrost Peatlands Remain Negligible After Wildfire and Thermokarst Disturbance. *J. Geophys. Res. Biogeosciences* **128**, e2022JG007322 (2023).
19. Voigt, C. *et al.* Arctic soil methane sink increases with drier conditions and higher ecosystem respiration. *Nat. Clim. Change* **13**, 1095–1104 (2023).
20. Kuhn, M. A. *et al.* BAWLD-CH4: a comprehensive dataset of methane fluxes from boreal and arctic ecosystems. *Earth Syst. Sci. Data* **13**, 5151–5189 (2021).
21. Olefeldt, D., Turetsky, M. R., Crill, P. M. & McGuire, A. D. Environmental and Physical Controls on Northern Terrestrial Methane Emissions Across Permafrost Zones. *Glob. Change Biol.* **19**, 589–603 (2013).
